# Treating Agricultural Runoff with a Mobile Carbon Filtration Unit

**DOI:** 10.1007/s00244-022-00925-8

**Published:** 2022-04-16

**Authors:** Bryn M. Phillips, Laura B. McCalla Fuller, Katie Siegler, Xin Deng, Ron S. Tjeerdema

**Affiliations:** 1 Department of Environmental Toxicology, Marine Pollution Studies Laboratory, University of California Davis, 34500 Highway One, Monterey, CA 93940 USA; 2 California Department of Pesticide Regulation, 1001 I Street, Sacramento, CA 95814 USA

## Abstract

Several classes of pesticides have been shown to impair water quality in California, including organophosphates, pyrethroids and neonicotinoids. Vegetative treatment systems (VTS) can reduce pesticide loads and associated toxicity in agricultural runoff, but many water-soluble pesticides such as neonicotinoids are not effectively treated by VTS, and VTS installation is not always an option for growers required to remove non-crop vegetation for food safety concerns. Recent studies have shown that biochar filtration can be used to remove soluble contaminants, especially when coupled with other VTS components. We evaluated a mobile carbon filter system consisting of a trailer-mounted tank containing approximately 600L (~ 180 kg) of biochar. Input water from a 437-hectare agricultural drainage was pre-filtered and treated with biochar during two multi-week study periods. Laboratory toxicity tests and chemical and nutrient analyses were conducted on input and output water. Pesticide concentrations were initially reduced by greater than 99%. Treatment efficacy declined linearly and was expected to remain at least 50% effective for up to 34 weeks. Toxicity was assessed with *Ceriodaphnia dubia*, *Hyalella azteca* and *Chironomus dilutus*. Significant input toxicity was reduced to non-toxic levels in 6 of 16 samples. Some input concentrations of the neonicotinoid imidacloprid and the pyrethroid cypermethrin exceeded organism-specific toxicity thresholds and benchmarks, but the overall causes of toxicity were complex mixtures of agricultural chemicals. Nutrients were not reduced by the biochar. Results demonstrate the utility of biochar in treating agricultural runoff and provide measures of the longevity of biochar under field conditions.

**Supplementary Information:**

The online version contains supplementary material available at 10.1007/s00244-022-00925-8.

Much of California’s intensive vegetable production, valued at over $7 billion annually, occurs in the Central Coast region of the state. Monterey and Santa Cruz counties produce many of the cool season vegetables in the coastal valleys, including lettuce, spinach and cole crops. Growers of these crops rely on several classes of pesticides to protect crops from insect pests, weeds and disease, and many of these chemicals enter waterways as runoff from agricultural operations. Several classes of pesticides used in vegetables have been shown to impair water quality in California, including organophosphates, pyrethroids and neonicotinoids (Central Coast Regional Water Quality Control Board 2011). Pesticides in runoff from irrigated vegetable production have been shown to cause a range of adverse impacts to aquatic ecosystems. These include acute toxicity to aquatic organisms (Anderson et al. 2014, 2018; Phillips et al. 2012; Schulz 2004), ecological impacts to invertebrate communities (Anderson et al. 2003, 2006b; Liess and von der Ohe, 2005), adverse impacts on salmonids (Scholz et al. 2000) and food web magnification in fish and wildlife (Pereira et al. 1996; Smalling et al. 2013).

On-farm treatment practices can be used to address chemical loads in irrigation runoff and are designed to treat pesticides with a range of solubilities (Cahn and Phillips 2019). Practices such as sediment basins, vegetation or the flocculent polyacrylamide not only reduce sediment load, but also concentrations of hydrophobic compounds such as pyrethroid insecticides and moderately hydrophobic compounds such as the organophosphate insecticide chlorpyrifos. Carbon treatment coupled with vegetation can reduce the load of more soluble pesticides, such as the organophosphate diazinon and the neonicotinoid imidacloprid.

Research has demonstrated that combining these practices into integrated vegetative treatment systems (VTS) reduces pesticide loads and associated toxicity in agriculture tailwater runoff. Sediment basins integrated in sequence with vegetated ditches within a VTS can reduce pyrethroid pesticides up to 100% (Anderson et al. 2011). The addition of compost and granulated activated carbon to a grass-lined ditch has been shown to reduce 98, 97 and 99% of the loads of the organophosphate pesticide chlorpyrifos (Phillips et al. 2017), the neonicotinoid imidacloprid and the pyrethroid permethrin, respectively (Phillips et al. 2021). These integrated systems have also been shown to reduce pesticide-associated toxicity to aquatic invertebrates in irrigation runoff.

Many water-soluble pesticides such as neonicotinoids are not effectively treated by VTS (Stang et al. 2016) and the use and detections of this class of insecticides are increasing in agricultural regions of California (Deng 2015; Deng et al. 2019). Imidacloprid is used in conjunction with pyrethroids on most lettuce crops in the Salinas Valley, and has recently been detected in irrigation runoff in the Central Coast region. Because neonicotinoids are water-soluble, they can be transported from application sites via surface water runoff and groundwater (Bonmatin et al. 2015). Activated carbon filtration is commonly used to remove organic compounds from wastewater and has been suggested for surface water treatment (Kalmykova et al. 2014; Pryor et al. 1999), while biochar has been used as remediation for contaminated soils (Jin et al. 2016; Yu et al. 2009). Recent research has been conducted applying biochar to treat pesticides in simulated agricultural runoff (Cederlund et al. 2017; Taha et al. 2014; Voorhees et al. 2020), but few studies assess the effectiveness of biochar-based water filter to reduce pesticide load and associated laboratory toxicity. Phillips et al. (2021) evaluated an integrated vegetated treatment system containing either granulated activated carbon or biochar. The system was tested with both simulated irrigation runoff spiked with permethrin and imidacloprid, as well as runoff from a conventionally grown lettuce field. Average reductions of permethrin and imidacloprid were 90%, and results were comparable between the two carbon materials (Phillips et al. 2021).

While carbon filtration has been shown to be effective at reducing contaminant concentrations and toxicity in vegetated ditches, installation of a VTS is not always an option for growers. On the intensively farmed Central Coast of California, acreage is often leased per growing season, with no incentive or ability to create permanent treatment systems on the property. Additionally, food safety concerns related to creation of potential habitat for bacterial vectors has discouraged some property owners and lessees from establishing vegetated BMPs (Baur et al. 2016). A mobile carbon filtration treatment system, either offered as a rented service or as a purchased product, could be used in lieu of a permanent installation.

The objective of this study was to evaluate the treatment effectiveness and longevity of a prototype mobile filtration unit (Leland Environmental Solutions, Walnut Creek CA), which was constructed to be a self-contained system utilizing particle filters and biochar. Originally developed for industrial wash water, this filter has potential for agricultural applications as a trailer-mounted unit situated proximate to a water source such as a sediment pond, drainage ditch or sump. Seven field trials consisting of laboratory toxicity tests and chemical and nutrient analyses of input and output water were conducted to assess how well the unit treats agricultural runoff over time. These data will provide information to estimate the lifetime of the filter media and the potential volume of water and chemical load the unit can treat. The treatment system will be evaluated for estimated costs and potential benefits. Results will serve as a guide to aid in management decisions related to the installation of a mobile carbon filtration management practice.

## Methods

**Fig. 1:**
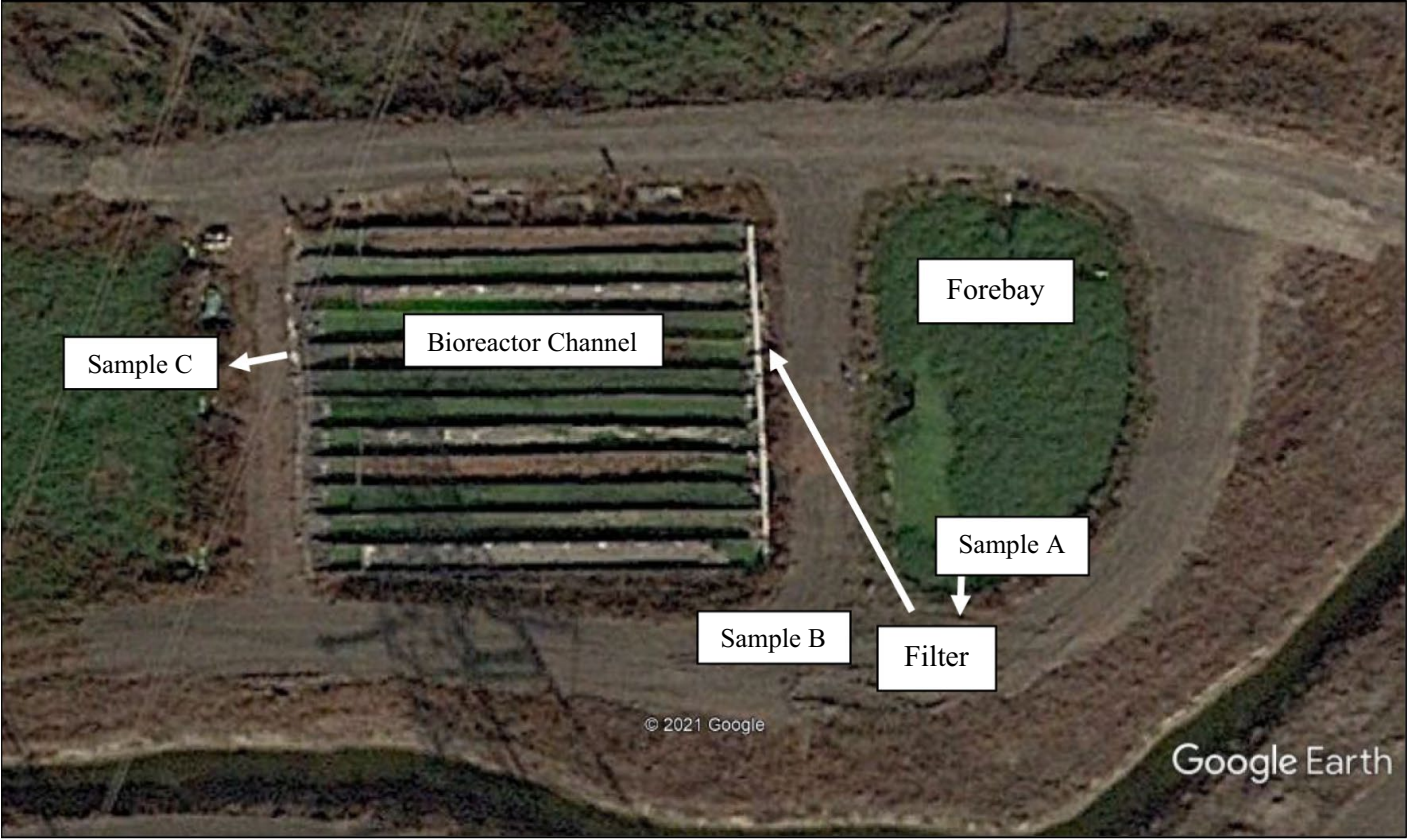
Aerial view of forebay and multi-channel bioreactor, and locations of mobile filter and sampling locations. Arrows indicate water flow from forebay, through the filter, through the bioreactor channel, and into the wetland

Field trials were conducted at an experimental multi-channel bioreactor and wetland administered by the Central Coast Wetlands Group (Krone et al. 2022) located in Castroville, CA (Fig. 1). The forebay that provides water to the bioreactor channels served as the input source for the mobile filter. This bioreactor forebay receives runoff from the Castroville Ditch, which receives water from 487 hectares of irrigated farmland with vegetable production that includes artichokes, Brussels sprouts, lettuce, and celery. Runoff is known to contain a variety of agricultural chemicals. Irrigation runoff was pumped from the forebay and through the filtration unit at a maximum rate of approximately 12L/minute. This rate was chosen to be similar to the rates normally used in the bioreactor system. Effluent from the filtration unit flowed into a woodchip bioreactor channel to be further treated for nutrients and to potentially have the wood chips provide further treatment for pesticides.

**Fig. 2:**
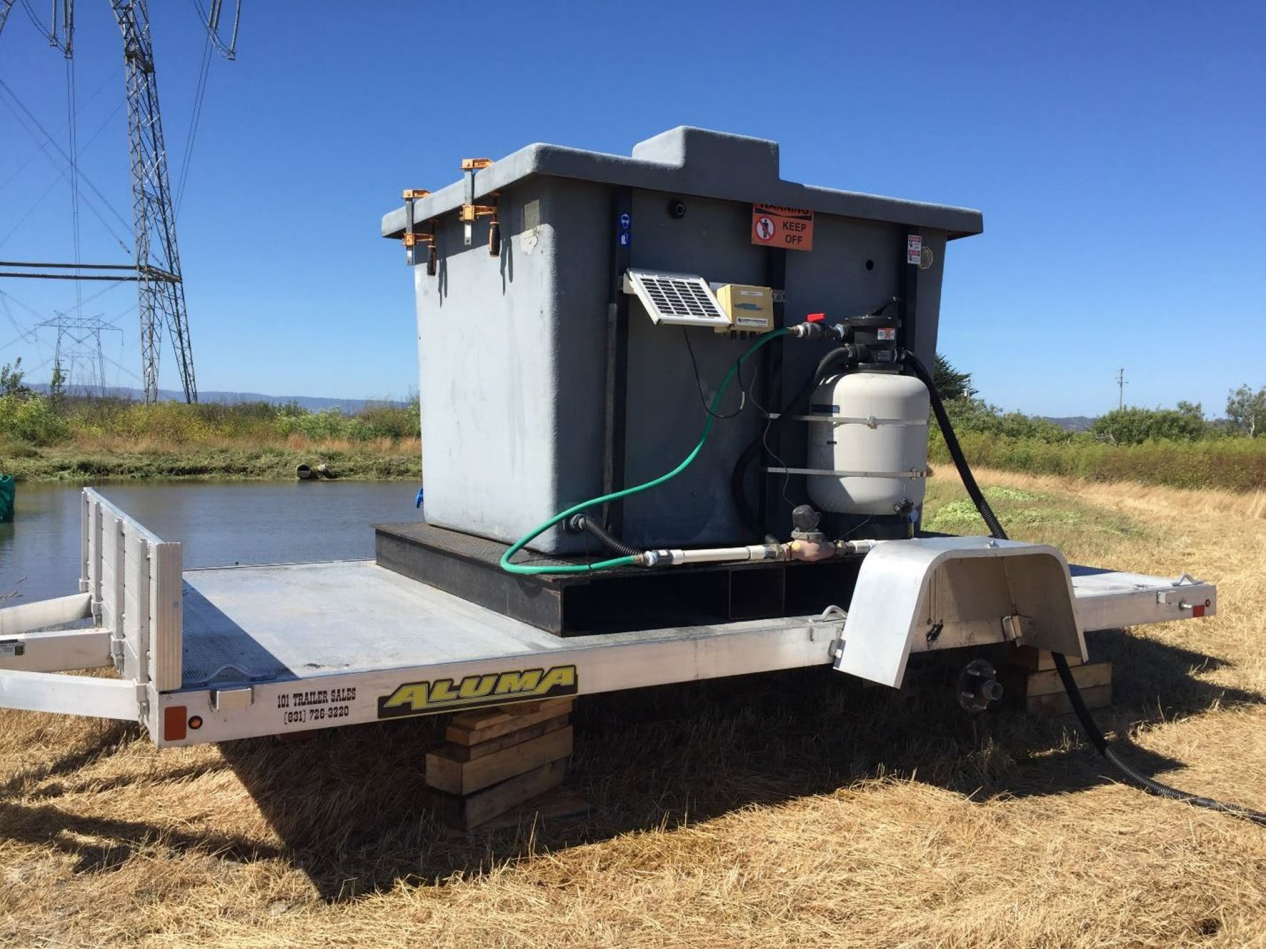
Trailer-mounted mobile filter. Forebay pictured in background

The mobile filter consisted of a fiberglass tank (Fig. 2) filled with approximately 600L (~ 180 kg) of biochar derived from organic, sustainably grown yellow pine wood pyrolyzed at 900 °C (AgraMarketing, Tracy CA). Input water entered the filter via a submersible pump and hose and first passed through a filter (Hayward Industries, Berkeley Heights, New Jersey) containing 45 kg of #35 sand before being diffused across the surface of the biochar. The sand filter was backwashed approximately three times per week. Water percolated through the biochar and exited the bottom of the tank. Filtered water then ran through a 5-cm hose to the input of a single bioreactor channel. The channel contained approximately 18 cubic meters of pine and oak wood chips with a length of 23 m, width of 2 m and a depth of 0.5 m. Wood chips varied in size from approximately 0.2 to 2 cm wide and 3 to 7 cm long. Residence time in the reactor channel was estimated to be approximately 24 h and there was no surface flow (Krone et al. 2022).

Twenty-four hour composite water samples from the forebay (A), the filter output (B) and the bioreactor output (C) were collected approximately every three weeks and were subsampled for toxicity and chemistry analysis (Fig. 1). Twenty-liter composite samples consisted of 800 mL subsamples collected hourly using a Global Water WS700 sampler (Global Water, College Station TX). Water filtration began on June 10, 2020, and continued through November 4, 2020, with minor interruptions. This schedule allowed for a range of upstream irrigation events to contribute runoff water with variable chemical concentrations to the system. One-liter subsamples were analyzed for a suite of insecticides, fungicides and herbicides by the California Department of Food and Agriculture Environmental Monitoring Laboratory (CDFA). Liquid chromatography/mass spectrometry (LC/MS) was used to measure 61 compounds, and gas chromatography/mass spectrometry (GC/MS) was used to measure six pyrethroid insecticides listed in Table S1 in Supplemental Information. Daily flows through the filter were monitored with a mechanical flowmeter (Netafim, Fresno, CA USA) attached to a datalogger (CR206x, Campbell Scientific, Logan, UT USA). Average daily discharge data were used to calculate average load reductions for every three-week sampling period.

Each sample underwent toxicity testing using acute exposures with the amphipod *Hyalella azteca* (96 h), the daphnid *Ceriodaphnia dubia* (96 h), and chronic exposures with the midge *Chironomus dilutus* (10 days). Organisms were chosen based on their varying sensitivities to agricultural chemicals. Testing procedures for the amphipod and daphnid followed U.S. EPA methodology (U.S. EPA 2002), and methods for the midge were based on USGS methods for the chronic exposure (Ingersoll et al. 2013; Kunz et al. 2017). Significant toxicity was determined statistically using a separate-variance t-test followed by a comparison to a threshold of 80% of the control response. To identify major causes of toxicity, organism responses were compared to the median lethal concentrations of detected chemicals (LC50s), as well as U.S. EPA Aquatic Life Benchmarks listed in Table S2 in Supplemental Information.

## Results and Discussion

### Water Treatment

**Fig. 3:**
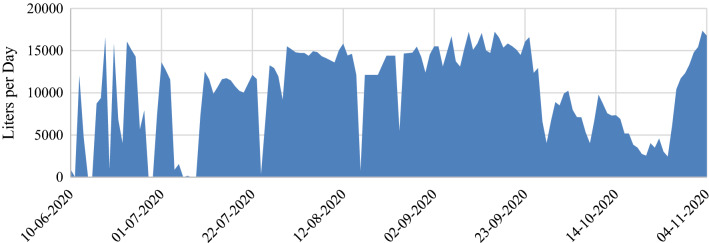
Daily filtration rates. Dates indicate three-week sampling periods. Nine-week trial ended and 12-week trial began on 8/12/2020

During the study period, there were minor interruptions due to power outages, forebay pump malfunctions, lack of water in the forebay, and filter clogging; but over 1.5 million liters of water passed through the filter, an average of over 10,000 L per day. Various pumping interruptions and slowdowns affected the volume of water filtered during each sampling period (Fig. 3). The original goal was to use the same batch of biochar continuously throughout the study, but because of heavy particle loads in the input water, the initial batch of biochar began to clog during the third period after approximately eight weeks. Although the nominal size of the sand in the pre-filter was 0.5 mm, fine particles still occluded the biochar and slowed the water flow. Reduced flow did not seem to reduce treatment efficacy, but additional pre-filters were necessary to maintain adequate flow for the bioreactor channel. New biochar and 100-µm glass fiber particle filters were installed after the third sampling event, effectively dividing the study into a nine-week trial and a twelve-week trial. Because of excessive particle loads in the forebay, filters were changed three times per week when the sand filter was backwashed.

### Chemistry

All analytical laboratory blanks were non-detect, and recovery of laboratory control material spikes and surrogate spikes were within acceptable limits (62.3%—120%) with the exception of clothianidin. Clothianidin recoveries ranged from 44.0% to 59.6% and were considered qualitative, but maintained in the data set to demonstrate removal through filtration. Analytical results from the fifth sampling period were also omitted because errors in sample handling rendered the data unusable.

A range of 11 to 21 compounds were detected in each sampling period (Table 1). Throughout the study, fifteen insecticides were detected, as well as eight fungicides and three herbicides. Some compounds were detected in every event, while others were only detected in a single event. Neonicotinoid insecticides were measured at the highest concentrations, followed by the fungicide mefenoxam and the herbicide bensulide.

**Table 1:** Concentrations of detected chemicals in pre- and post-filtration samples (A and B, respectively), and calculation of percent reduction through treatment

Analyte (ng/L)	Type	Period 1A (7/1/20)			Period 1B (7/22/20)			Period 1C (8/12/20)			Period 2A (9/2/20)			Period 2C (10/14/20–6 weeks)			Period 2D (11/4/20)		
		A	B	% Red	A	B	% Red	A	B	% Red	A	B	% Red	A	B	% Red	A	B	% Red
Acetamiprid (µg/L)	Neonicotinoid										0.091	ND	− 100						
Clothianidin (µg/L)	Neonicotinoid	*0.425*	ND	− 100	*2.37*	*0.062*	− 97	*1.04*	0.023	− 97.8	*0.550*	ND	− 100	*0.547*	ND	− 100	*0.815*	ND	− 100
Imidacloprid (µg/L)	Neonicotinoid	*0.481*	ND	− 100	*1.42*	*0.040*	− 97	*0.942*	*0.024*	− 97.5	*1.35*	*0.013*	− 99.1	*3.97*	ND	− 100	1.73	0.148	− 91.4
Thiamethoxam (µg/L)	Neonicotinoid	*1.62*	0.026	− 98	*0.807*	*0.124*	− 85	*1.56*	0.201	− 87.1	*2.46*	0.043	− 98.2	*1.17*	0.049	− 95.8	0.128	0.054	− 58.0
Dimethoate (µg/L)	Organophosphate				0.034	ND	− 100												
Malathion (µg/L)	Organophosphate	0.030	ND	− 100							*0.070*	ND	− 100						
Bifenthrin (ng/L)	Pyrethroid	*1.04*	ND	− 100	*2.12*	ND	− 100	*2.03*	ND	− 100	*2.60*	ND	− 100	*3.66*	ND	− 100	*1.88*	ND	− 100
Cypermethrin (ng/L)	Pyrethroid							*581*	ND	− 100	*6.94*	ND	− 100	1.04	ND	− 100			
Etofenprox (ng/L)	Pyrethroid										100	ND	− 100						
Permethrin (ng/L)	Pyrethroid				*3.60*	ND	− 100	*5.29*	ND	− 100	*10.2*	ND	− 100						
Chlorantraniliprole (µg/L)	Ryanoid	0.480	ND	− 100	0.377	0.066	− 83	1.22	0.157	− 87.1	1.59	0.032	− 97.9	1.01	ND	− 100	0.327	0.099	− 69.8
Fipronil amide (µg/L)	Phenylpyrazole				0.011	ND	− 100												
Methomyl (µg/L)	Carbamate	0.102	ND	− 100	0.037	ND	− 100	0.165	ND	− 100	*2.00*	ND	− 100						
Methoxyfenozide (µg/L)	Diacylhydrazine	0.153	ND	− 100	0.128	0.050	− 61	0.176	0.059	− 66.5	0.167	ND	− 100	0.822	ND	− 100	0.125	0.104	− 16.8
Azoxystrobin (µg/L)	Fungicide	0.045	ND	− 100	0.045	ND	− 100	0.489	ND	− 100	0.142	ND	− 100	0.044	ND	− 100	0.044	ND	− 100
Boscalid (µg/L)	Fungicide	1.28	ND	− 100	0.699	0.025	− 96	0.534	0.023	− 95.8	0.499	ND	− 100	0.408	ND	− 100	0.330	ND	− 100
Fenamidone (µg/L)	Fungicide	0.031	ND	− 100										0.150	ND	− 100			
Fludioxonil (µg/L)	Fungicide							0.810	ND	− 100	0.613	ND	− 100				0.081	ND	− 100
Mefenoxam (µg/L)	Fungicide	0.068	ND	− 100	2.24	0.811	− 64	1.88	1.02	− 45.5	2.99	0.282	− 90.6	0.960	0.706	− 26.5	1.99	0.831	− 58.2
Propiconazole (µg/L)	Fungicide																		
Pyraclostrobin (µg/L)	Fungicide				0.102	ND	− 100	0.040	ND	− 100	0.101	ND	− 100						
Tebuconazole (µg/L)	Fungicide																		
Thiabendazole (µg/L)	Fungicide										0.023	ND	− 100						
Trifloxystrobin (µg/L)	Fungicide										0.045	ND	− 100						
Bensulide (µg/L)	Herbicide	0.063	ND	− 100	0.051	ND	− 100	6.14	0.045	− 99.3	0.082	ND	− 100						
Diuron (µg/L)	Herbicide	0.050	ND	− 100	0.026	ND	− 100				0.034	ND	− 100				0.028	ND	− 100
Prometryn (µg/L)	Herbicide				0.043	ND	− 100												
Mean reduction (%)				− 99.9			− 93.1			− 91.8			− 99.3			− 92.9			− 81.3
Volume treated (L)			172,464			193,207			274,517			269,530			438,442			153,635	
Load reduction (g)				0.75			1.39			3.85			3.38			3.43			0.31

Italic cells indicate an exceedance of an organism-specific LC50 or U.S. EPA Aquatic Life Benchmark. Blank cells indicate a chemical was not detected, and ND indicates non-detect in post-treatment sample

The first batch of biochar was used for nine weeks, during which time samples were collected three times. During the first three-week sampling period (1A), the filter system reduced twelve of the thirteen compounds detected in the input sample to below detection limits in the output sample, for an average treatment efficacy of 99.9% (Table 1). This efficacy began to wane in the second (1B) and third (1C) treatment periods. Eleven of seventeen compounds were reduced below detection limits by six weeks of treatment, and eight of fifteen compounds by nine weeks of treatment, for treatment efficacies of 93.1% and 91.8%, respectively. At this stage of the study, the biochar was getting increasingly clogged with fine particles and the water flow was getting significantly reduced.

The biochar was replaced with similar material, and 100-µm fine particle filters were added to the system for the remaining four sampling periods (2A–2D). During period 2A, the new biochar reduced concentrations of 17 of 21 compounds to below detection limits, for a treatment efficacy of 99.3%. Analytical results from the period 2B were unusable, but concentrations of nine of eleven compounds were reduced below detection limits during period 2C (92.9% efficacy), and the concentrations of five of eleven compounds during period 2D (81.3% efficacy).

Load is the total mass of pesticides calculated as a product of the measured concentrations and the water volume treated during each period. Concentrations presented in Table 1 are in microgram or nanogram per liter units, but when multiplied by the amount of water treated, grams of chemicals were removed by the filter. Load reductions ranged from 0.31 g to 3.85 g per sampling period and totaled over 13 g for the entire study.

### Toxicity

All toxicity tests met test acceptability criteria for minimum percent survival in the control (≥ 90% for *C. dubia* and *H. azteca*, ≥ 80% for *C. dilutus*, Table 2). Toxicity test organisms exposed to reference concentrations of salts and metals also responded as expected, indicating all test organisms were healthy and had the appropriate sensitivities during the exposures.

**Table 2:** Mean survival results summary for testing with the daphnid (*C. dubia*), amphipod (*H. azteca*) and midge (*C. dilutus*)

Sample	Period 1A (7/1/20)		Period 1B (7/22/20)		Period 1C (8/12/20)		Period 2A (9/2/20)		Period 2B (9/23/20)		Period 2C (10/14/20)		Period 2D (11/4/20)	
	Mean	SD	Mean	SD	Mean	SD	Mean	SD	Mean	SD	Mean	SD	Mean	SD
*C. dubia*	Survival (%)													
A	*0*	*0*	*20*	*24*	*0*	*0*	*0*	*0*	*0*	*0*	96	9	92	11
B	100	0	96	9	76	9	*72*	*11*	*48*	*18*	96	9	96	9
Control	100	0	100	0	92	18	100	0	100	0	100	0	96	10
*H. azteca*	Survival (%)													
A	93	7	*42*	*26*	*0*	*0*	*32*	*15*	96	5	96	5	*58*	*11*
B	100	0	98	4	*17*	*12*	100	0	98	4	100	0	96	9
Control	90	10	100	0	96	9	94	5	100	0	98	4	100	0
*C. dilutus*	Survival (%)													
A	*0*	*0*	*0*	*0*	*0*	*0*	*0*	*0*	*0*	*0*	*0*	*0*	*0*	*0*
B	*58*	*23*	*0*	*0*	*0*	*0*	*0*	*0*	*0*	*0*	*71*	*20*	*0*	*0*
Control	83	10	90	8	96	8	92	7	92	7	100	0	90	8
*C. dilutus*	Growth (mg/ind.)													
A	NA	NA	NA	NA	NA	NA	NA	NA	NA	NA	NA	NA	NA	NA
B	*0.35*	*0.12*	NA	NA	NA	NA	NA	NA	*0.10*	*NA*	0.76	0.54	NA	NA
Control	0.93	0.47	0.88	0.61	3.66	1.88	1.83	1.04	3.84	0.57	1.11	0.48	1.97	1.49

Samples were collected from the forebay (A) and post-filter (B). Control indicates negative dilution water control used in toxicity tests. Italic indicates significant toxicity. SD indicates standard deviation. NA indicates not analyzed

Filter input samples from the forebay were all significantly toxic to at least one test organism during every sampling event. Period 1C was the most toxic with no survival of any of the three organisms (Table 2). *Chironomus dilutus* was the most sensitive organism to the input water, with complete mortality observed in every input sample. Five of seven input samples were significantly toxic to *C. dubia*, and four of seven were significantly toxic to *H. azteca*. All five input samples toxic to *C. dubia* received significant treatment with the filter. Two post-filter samples were still significantly toxic, but the filter increased *C. dubia* survival from an average of 4% in the input samples to 78.4% in the post-filter samples. Only one input sample caused complete mortality to *H. azteca* (Period 1C), but within the four toxic input samples, the filter increased average *H. azteca* survival from 33 to 77.8%. One post-filter sample was still significantly toxic to *H. azteca*. Although *C. dilutus* was the most sensitive organism, the filter improved survival during two periods, but these post-filter samples were still considered significantly toxic. Across all of the sampling periods, the filter improved average *C. dilutus* survival from 0 to 18.7%. Because there were no *C. dilutus* survivors in the input samples, it was impossible to calculate improvements to midge growth, but growth in the post-filter sample from Period 2C was not significantly different from the control, indicating the filter had also removed chronic toxicity. Input samples caused significant toxicity in sixteen of the twenty-one separate toxicity tests that were conducted. The filter increased the overall survival from 12.3% to 58.3%, and significant toxicity was completely removed in six post-filter samples.

There was no single chemical class that caused all of the observed toxicity, but some concentrations of the neonicotinoid imidacloprid and the pyrethroid cypermethrin exceeded organism-specific toxicity thresholds and benchmarks (Table S2). The imidacloprid LC50 for *C. dilutus* was exceeded in forebay samples collected during periods 2C and 2D. The filter completely removed imidacloprid in period 2C and removed over 90% of this insecticide in period 2D. Period 2C saw the best recovery of *C. dilutus* survival in the post-filter sample (Table 2). Cypermethrin LC50s for both *H. azteca* and *C. dilutus* were exceeded during periods 1C and 2A, with the cypermethrin concentration in period 1C exceeding the *H. azteca* LC50 by over 250 times. The filter also reduced these concentrations to non-detectable levels. Pyrethroids are hydrophobic contaminants that readily associate with surfaces and are therefore easier to remove with carbon filter media than more soluble compounds, such as imidacloprid.

Although only two insecticides exceeded organism-specific LC50 values, seven insecticides exceeded the U.S. EPA Aquatic Life Benchmarks (Table 2). Benchmark concentrations are generally more conservative than median lethal concentrations, but an exceedance of these values can still indicate possible contributions to toxicity. The neonicotinoids clothianidin, imidacloprid and thiamethoxam, as well as malathion, bifenthrin, permethrin and methomyl all exceeded their respective chronic benchmarks for invertebrates. Imidacloprid exceeded its acute invertebrate benchmark. Aquatic life benchmarks are unlike LC50 thresholds in that they are more protective and likely more indicative of ecosystem health impacts. Benchmark concentrations are estimates below which pesticides are not expected to represent a risk for aquatic life. Input samples from the forebay exceeded benchmark values during every sampling period, but the filter was able to reduce concentrations below benchmark values in 75% of these samples.

The bioreactor forebay, which supplied water to the filter, received agricultural runoff from over 437 hectares. Crops on these fields used a variety of pesticides for the treatment of insects, fungi and weeds. It is the mixture of these compounds that ultimately caused the toxicity. Although CDFA was able to measure 61 compounds, this analyte list was still limited compared to the number of compounds applied in the watershed. Toxicity of some compounds, such as pyrethroids, can be additive because they have the same mode of action, and this additivity can be calculated. For example, a toxic unit approach can be used to calculate the relative contribution of each pyrethroid based on the measured concentration divided by the LC50 value. A single toxic unit would be expected to cause 50% mortality. The sum of pyrethroid toxic units for *H. azteca* explains three of the four significant reductions of survival. Much of the other observed toxicity cannot be easily explained, but is clearly caused by the mixture of compounds draining to the watershed. Complex mixtures are the main reason that conducting toxicity testing is imperative to provide a quantitative measurement and to determine potential impacts on the receiving system.

### Bioreactor Treatment

The main purpose of the wood chip bioreactor was to provide a substrate for bacteria to reduce nutrients through denitrification or other biochemical processes (Krone et al. 2022). Post-filter water was passed through a single channel of wood chips to provide additional treatment for nutrients, and to determine if the wood chips would provide additional pesticide removal. The average concentration of nitrate in the forebay (Table 3, Sample A) was 26.1 mg/L, and the average concentration in the post-filter samples was 27.5 mg/L, indicating no nutrient reduction took place in the filter. Although biochar can reduce nitrogen concentrations give longer contact time (Saarela et al. 2020), nitrate is extremely soluble and was not expected to bind to the carbon during the short residence period in the filter. The processes that reduce nitrate in the wood chip bioreactor require bacteria, heat and residence time, which were not feasible with the small biochar filter. Average concentrations at the downstream end of the wood chip channel were 5.6 mg/L, indicating a 79% reduction in nitrate.

**Table 3:** Nitrate concentrations (mg/L) measured in samples from the forebay (A), post-filter (B) and post-wood chips (C)

Sample	Period 1A (7/1/20)	Period 1B (7/22/20)	Period 1C (8/12/20)	Period 2A (9/2/20)	Period 2B (9/23/20)	Period 2C (10/14/20)	Period 2D (11/4/20)
A	25.7	38.4	18.0	29.0	39.0	28.0	17.3
B	24.0	39.8	18.2	26.5	35.8	39.4	17.3
C	1.80	7.60	9.30	9.40	4.60	4.40	1.10
Percent reduction	− 93.0	− 80.2	− 48.3	− 67.6	− 88.2	− 84.3	− 93.6

In most cases, the wood chip channel did not further reduce contaminant concentrations, but added pesticides back to the post-filter input water (data not shown). For example, during period 1A, the concentrations of all but one compound were reduced to below detection limits. Only thiamethoxam was not completely removed (Table 1). Of the thirteen compounds detected in the forebay input water, twelve were non-detects in the post-filter sample, but seven were again detected in the post wood chip sample. Clothianidin and azoxystrobin were detected at concentrations higher than those detected in the forebay, and two other compounds, propiconazole and prometryn had not been detected in the forebay, but were detected downstream of the wood chips. There was still a net reduction of chemical concentrations between sample A and sample C, but the return of compounds by the wood chips indicates the bioreactor can be a source of previously bound contaminants if uncontaminated water is used for the input.

Several samples of water that had passed through the wood chips had high enough concentrations of pesticides added back to them to cause significant toxicity (data not shown). Periods 1B, 1C and 2B had significant treatment of toxicity to *C. dubia* in the post-filter sample and had toxicity returned in the post wood chip sample. This was also true of *H. azteca* in period 1C. Significant treatment of C. dilutus toxicity only occurred during periods 1A and 2C, but complete mortality was observed in all post wood chip samples.

The wood chips in this system had been put in place to treat nitrate in the spring of 2017. Since that time, the bioreactor has processed millions of liters of water and continues to successfully reduce nitrate concentrations. The wood chips have also served as a substrate and carbon source for the binding of agricultural chemicals, and these chemicals appear to be leaching from the wood chips into the clean water that is passing through the channel. This situation might have been alleviated by placing the biochar filter downstream of the wood chip channel, but the primary objective of this study was to determine the raw treatment efficacy of the filter as a standalone unit. It was assumed that the bioreactor would serve its purpose with nitrate reduction, but it also ultimately lessened the overall load reduction.

### Longevity

It is difficult to determine how long a batch of carbon will successfully treat agricultural runoff. Whether the filter media are granulated activated carbon or biochar, there are several factors that will affect carbon efficacy, including water volume, flow rate, contaminant load and particle load. The lower threshold of acceptable treatment that will trigger replacing the carbon needs to be determined according to individual treatment goals. In the case of granulated activated carbon, the earliest breakthrough of contaminants into the effluent might indicate replacement (U.S. EPA 1991), but with biochar use in an agricultural setting, it would be costly and difficult to constantly monitor chemical concentrations downstream of the carbon treatment. A more likely solution would be to over-engineer the system for a worst-case scenario, safe in the knowledge that the filter could last for a known period. In the case of the filter used in this study, multiple units could be placed in parallel to increase capacity.

Fouling of the biochar with particles caused this study to be divided into two treatment efficacy study periods. This situation effectively created two experimental replicates. The waning treatment efficacy from both periods was similar. The first period lasted nine weeks and saw the efficacy go from 99.9% to 91.8%. During the second period of twelve weeks, the efficacy went from 99.3% to 81.3%. The downward efficacy slope of each study period was approximately − 1.6, indicating that barring additional complications from clogging, the biochar used in either treatment period would have lasted approximately 34 weeks under similar irrigation volumes before reaching 50% efficacy, and approximately 58 weeks to reach 10% efficacy.

Creating a filter with a larger biochar bed would increase the efficacy and longevity. A larger system could also allow for a mixture of other materials with the biochar to increase flow and decrease clogging. Recent experiments with mixtures of biochar and pumice show increased flow efficiency (unpublished data), and the use of biochar and wood chip mixtures in recharge basins have demonstrated increased infiltration efficiency over biochar alone (Andrew Fisher, UC Santa Cruz, personal communication). Further study is needed to determine an optimal mixture of flow and treatment, but current studies show that if water can come in contract with the biochar, treatment will occur (Phillips et al. 2021).

### ***Cost–***Benefit Analysis

Understanding the economic advantages and disadvantages of this type of management practice will be helpful in evaluating management decisions related to this technology. This type of practice can be small and portable and could potentially be used in a variety of locations either individually, in series or in parallel. Initial cost and maintenance will vary based on need, but improved water quality will reduce economic grower liability for producers under regulatory frameworks addressing nonpoint source runoff. This management practice could potentially be added to the list of practices recommended and subsidized by agencies like the USDA Natural Resources Conservation Service (NRCS) through their Environmental Quality Incentives Program (EQIP), or through grant funded projects by Resource Conservation Districts or local agencies, thus providing economic incentives for installation.

The current practice utilized 600 L (~ 180 kg) of biochar in a specialized filter housing, but costs could be minimized by using a similar volume of biochar in less expensive, and more easily manageable 55-gallon drums. Using locally sourced biochar prepared from a variety of materials could reduce costs. Ideally, particle-free runoff will be pumped from a sediment trap or pond, but if the water source contains a heavy particle load, optional sand and glass-fiber filters can be used to reduce suspended particles. Open-topped plastic drums would be plumbed with diffusers to distribute water over the biochar mixture. Water would be collected from the bottom of the biochar column and conveyed to a drainage.

Producers of diverse crops can benefit from carbon filtration of their runoff. Improved water quality in tailwater runoff will lead to reductions of pesticide concentrations and occurrences of pesticide-related toxicity. Reductions in pesticide loads will promote healthier invertebrate communities, with cascading benefits to fish, birds, and other wildlife in extended food webs. Migratory birds, wetland habitats, and anadromous fish are all expected to benefit directly and/or indirectly from reduced pesticide inputs to waterways. Use of the filter for treating agricultural runoff may also be attractive to growers previously unable to commit to established conservation practices. A mobile treatment system, either offered as a rented service or as a purchased product, could be used on properties without the ability to install vegetated treatment systems, or as a final step in an integrated system to remove multiple classes of pesticides. Use of a filter system for treating agricultural runoff will not promote bacterial vectors associated with food safety concerns. Potential drawbacks of using the filter system are unknown costs, particularly in the form of maintenance. Variable conditions on a farm might lead to excessive particle loads in the input water, which would lead to increased labor and materials costs for keeping the pre-filters clean and preventing the biochar from clogging. Local users and advisors can determine the required maintenance to adequately suit their treatment goals.

The results of this study demonstrated that a simple biochar filter can greatly reduce pesticide loads and related toxicity from real-world agricultural runoff. This study also demonstrated how long this particular biochar could effectively treat runoff, and the potential difficulties of managing this type of filter system. Although the system used in this study was repurposed for treating agricultural runoff, the technology could be easily adapted using lower-cost parts that are easily obtainable.

## Supplementary Information

Below is the link to the electronic supplementary material.

Supplementary file1 (DOCX 33 kb)

## Data Availability

The datasets generated and analyzed during the current study are available from the corresponding author on reasonable request. Not applicable.
