# Supplementary material for: Treating Agricultural Runoff with a Mobile Carbon Filtration Unit
Source: Arch Environ Contam Toxicol. 2022 Apr 16;82(4):455–66. doi: 10.1007/s00244-022-00925-8 (PMC9079026; doi:10.1007/s00244-022-00925-8)
Supplement: Supplementary file 1 — Supplementary file1 (DOCX 33 kb) [file 244_2022_925_MOESM1_ESM.docx]

# Supplemental Information

Table S1. Analyte list as associated reporting limits (RL) and method detection limits (MDL).

| **Analyte** | **RL** | **MDL** | **Analyte** | **RL** | **MDL** |
| --- | --- | --- | --- | --- | --- |
|  | **(µg/L)** | **(µg/L)** |  | **(µg/L)** | **(µg/L)** |
| Abamectin | 0.02 | 0.004 | Oxadiazon | 0.02 | 0.004 |
| Acetamiprid | 0.02 | 0.004 | Prometon | 0.02 | 0.004 |
| Atrazine | 0.02 | 0.004 | Prometryn | 0.02 | 0.004 |
| Azoxystrobin | 0.02 | 0.004 | Propanil | 0.02 | 0.004 |
| Bensulide | 0.02 | 0.004 | Propargite | 0.02 | 0.004 |
| Boscalid | 0.02 | 0.004 | Propiconazole | 0.02 | 0.004 |
| Bromacil | 0.02 | 0.004 | Pyraclostrobin | 0.02 | 0.004 |
| Carbaryl | 0.02 | 0.004 | Pyriproxyfen | 0.015 | 0.004 |
| Chlorantraniliprole | 0.02 | 0.004 | Quinoxyfen | 0.02 | 0.004 |
| Chlorpyrifos | 0.02 | 0.004 | Simazine | 0.02 | 0.004 |
| Clothianidin | 0.02 | 0.004 | S-Metolachlor | 0.02 | 0.004 |
| Cyprodinil | 0.02 | 0.004 | Tebuconazole | 0.02 | 0.004 |
| Diazinon | 0.02 | 0.004 | Tebufenozide | 0.02 | 0.004 |
| Diflubenzuron | 0.02 | 0.004 | Tebuthiuron | 0.02 | 0.004 |
| Dimethoate | 0.02 | 0.004 | Thiabendazole | 0.02 | 0.004 |
| Diuron | 0.02 | 0.004 | Thiacloprid | 0.02 | 0.004 |
| Ethoprop | 0.02 | 0.004 | Thiamethoxam | 0.02 | 0.004 |
| Etofenprox | 0.02 | 0.004 | Thiobencarb | 0.02 | 0.004 |
| Fenamidone | 0.02 | 0.004 | Trifloxystrobin | 0.02 | 0.004 |
| Fenhexamid | 0.02 | 0.005 | Atrazine-d5 | 0.02 | 0.004 |
| Fludioxonil | 0.02 | 0.004 | Imidacloprid-d4 | 0.02 | 0.004 |
| Hexazinone | 0.02 | 0.004 | Fipronil | 0.01 | 0.004 |
| Imidacloprid | 0.01 | 0.004 | Fipronil Amide | 0.01 | 0.004 |
| Indoxacarb | 0.02 | 0.004 | Fipronil Sulfide | 0.01 | 0.004 |
| Isoxaben | 0.02 | 0.004 | Fipronil Sulfone | 0.01 | 0.004 |
| Kresoxim-methyl | 0.02 | 0.004 | Desulfinyl Fipronil | 0.01 | 0.004 |
| Malathion | 0.02 | 0.004 | Desulfinyl Fipronil Amide | 0.01 | 0.004 |
| Mefenoxam | 0.02 | 0.004 | Bifenthrin | 0.001 | 0.00099 |
| Methidathion | 0.02 | 0.004 | Lambda Cyhalothrin | 0.002 | 0.00137 |
| Methomyl | 0.02 | 0.004 | Permethrin Cis | 0.001 | 0.00074 |
| Methoxyfenozide | 0.02 | 0.004 | Permethrin Trans | 0.001 | 0.00087 |
| Metribuzin | 0.02 | 0.004 | Cyfluthrin | 0.002 | 0.00200 |
| Norflurazon | 0.02 | 0.004 | Cypermethrin | 0.005 | 0.00183 |
| Oryzalin | 0.02 | 0.004 | Esfenvalerate/Fenvalerate | 0.005 | 0.00238 |

Table S2. Median lethal concentrations (LC50s) and U.S. EPA Office of Pesticide Programs Aquatic Life Benchmark concentrations used to evaluate detected chemicals in pre- and post-treatment samples (<https://www.epa.gov/pesticide-science-and-assessing-pesticide-risks/aquatic-life-benchmarks-and-ecological-risk>).

|  | ***C. dubia***  **LC50** | ***H. azteca***  **LC50** | ***C. dilutus***  **LC50** | **U.S. EPA Acute Benchmark** | **U.S. EPA Chronic Benchmark** |
| --- | --- | --- | --- | --- | --- |
| **Neonicotinoids** | **µg/L** | **µg/L** | **µg/L** | **µg/L** | **µg/L** |
| Acetamiprid | >33,500 (Raby et al. 2018) 96h LC50 | 4.8 (Raby et al. 2018) 96h LC50 | 2.8 (Raby et al. 2018) 96h LC50 | 10.5 | 2.1 |
| Clothianidin | >100,000 (Raby et al. 2018) 96h LC50 | 5.2 (Raby et al. 2018) 96h LC50 | 5.93 (Maloney et al. 2017) 96h LC50 | 11 | 0.05 |
| Imidacloprid | 72,124 (Raby et al. 2018) 96h LC50 | 65.4 (Stoughton et al. 2008) 96h LC50 | 1.52 (Cavallaro 2017) 14d LC50 | 0.385 | 0.01 |
| Thiamethoxam | >80,000 (Raby et al. 2018) 96h LC50 | 801 (Raby et al. 2018) 96h LC50 | 23.6 (Cavallaro 2017) 14d LC50 | 17.5 | 0.74 |
| **Organophosphates** | **µg/L** | **µg/L** | **µg/L** | **µg/L** | **µg/L** |
| Dimethoate | None | None | 1,290 (LeBlanc et al. 2012) 96h LC50 | 21.5 | 0.5 |
| Malathion | 2.120 (Ankley et al. 1991) 48h LC50 | 0.19 (Cothran et al. 2009) 96h LC50 | 0.62 (Hansen and Kawatski 1976) 96h LC50 | 0.049 | 0.06 |
| **Pyrethroids** | **ng/L** | **ng/L** | **ng/L** | **ng/L** | **ng/L** |
| Bifenthrin | 142 (Wheelock et al. 2004) 48h LC50 | 7.7 (Weston and Jackson 2009) 96h LC50 | 23 (Ding et al. 2012) 10d LC50 | 800 | 1.3 |
| Cypermethrin | 683 (Wheelock et al. 2004) 48h LC50 | 2.3 (Weston and Jackson 2009) 96h LC50 | 6.9 (Maund et al. 2002) 96h LC50 | 210 | 6.9 |
| Etofenprox | None | None | 16,900 (Yoshimi et al. 2002) 48h LC50 | 400 | 170 |
| Permethrin | 250 (Wheelock et al. 2004) 48h LC50 | 21.1 (Anderson et al. 2006) 96h LC50 | 99 (Ding et al. 2012) 10d LC50 | 19.5 | 1.4 |
| **Other Insecticides** | **µg/L** | **µg/L** | **µg/L** | **µg/L** | **µg/L** |
| Chlorantraniliprole | None | None | 4 (Maloney et al. 2020) 96h LC50 | 5.8 | 4.47 |
| Fipronil Amide | None | None | None | NA | NA |
| Methomyl | None | None | None | 2.5 | 0.7 |
| Methoxyfenozide | None | None | 620 (Smagghe et al. 2002) 120h LC50 | 28.5 | 3.1 |
| **Fungicides** | **µg/L** | **µg/L** | **µg/L** | **µg/L** | **µg/L** |
| Azoxystrobin | None | None | None | 130 | 44 |
| Boscalid | None | None | None | < 2,665 | 790 |
| Fenamidone | None | None | None | 24.5 | 12.5 |
| Fludioxonil | None | None | None | 450 | 14 |
| Mefenoxam | None | None | None | 26,900 | 1,200 |
| Pyraclostrobin | None | None | None | 7.85 | 4 |
| Thiabendazole | None | None | None | 155 | 42 |
| Trifloxystrobin | None | None | None | 12.65 | 2.76 |
| **Herbicides** | **µg/L** | **µg/L** | **µg/L** | **µg/L** | **µg/L** |
| Bensulide | None | None | None | 290 | 11 |
| Diuron | None | 19,400 (Nebeker and Schuytema 1998) 96h LC50 | 3,300 (Nebeker and Schuytema 1998) 10d LC50 | 80 | 200 |
| Prometryn | None | None | None | 4,850 | 1,000 |
